# Emergency medicine and global health policy: history and next steps

**DOI:** 10.7189/jogh.06.020304

**Published:** 2016-12

**Authors:** Stephen C. Morris

**Affiliations:** Division of Emergency Medicine, Department of Medicine, University of Washington School of Medicine, WA, USA

The transformation of global health and the specialty of emergency medicine are both relatively new phenomena. Over the last three decades, they have developed rapidly and in parallel. Utilizing a review of the recent changes of global health policy and practice, and a review of the changing nature and understanding of the global burden of disease and its intersection with emergency medical care, this paper attempts to demonstrate missed opportunities to support the development of emergency medicine on a global scale. This paper also seeks to highlight the need for improved alignment between the global burden of disease, global health policy–setting, and emergency medicine development.

Emergency medicine is a relatively new medical specialty that, when implemented, provides rapid management of acute critical illness and injury. The specialty of emergency medicine allows health care providers to adjust their scope of practice to respond to both the immediate situation and the long–term needs of their community. It supports other branches of medicine by freeing them to practice their own specialized skill set, by providing emergent supportive care, and by serving as a directed gateway to the most appropriate levels of health care delivery.

Regions that have weak or dysfunctional emergency medical services, or have not yet established emergency medicine as a specialty, are at risk for avoidable morbidity and mortality. The resulting impact on individual and population health creates an avoidable social and economic burden. Global burden of disease projections indicate that the developing world should expect more trauma and injury, higher rates of chronic illness, and changes related to urbanization [1]. Yet global health priority setting and funding allocations have failed to address the predicted increased need for emergency medical services. The global community has an opportunity and a responsibility to prioritize emergency medical care through policy changes and investment in education, awareness, and infrastructure to meet the needs of our evolving health care environment.

## RECENT GLOBAL HEALTH POLICY AND PRACTICE AND EMERGENCY MEDICINE

Highlighting some recent developments in global health policy demonstrate disconnect between global health care needs and resource allocation.

The Millennium Development Goals (MDGs) (2000) had potential through global governance to guide funding and policy but has also resulted in unbalanced aid and development. MDGs related to emergency medicine include: MDG 1: eradicate extreme poverty and hunger, MDG 3: promote gender equality and empower women, MDG 4: reduce child mortality, MDG 5: improve maternal health, MDG 6: combat HIV/AIDS, malaria and other diseases, MDG 7: ensure environmental sustainability and MDG 8: develop a global partnership for development. However projects and funding have not been allocated in response to burden or need. For example: Concerning MDG 6 an analysis of “official government assistance funding (ODA),” MDG 6 constituted approximately 50% of all health expenditure (1999–2009) with one half of that money going to just 10 countries representing only 21% of the population of least developed countries. In addition, MDG 5, represent only 10% of official development assistance, far below the actual burden of maternal related health expense.

**Figure:**
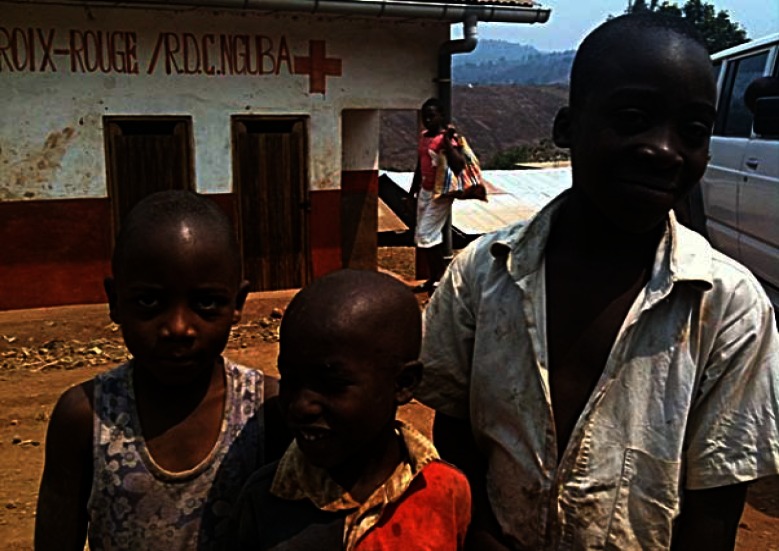
Photo: Courtesy of the author

The attention garnered by the millennium development goals, while robust, has removed emphasis from more rational health system development and policy, of which emergency medical care is an integral component. Greater emphasis on burden of disease, population and individual health, and health as a human right would represent more just and appropriate policy and resource utilization.

Elements of emergency care such as trauma and treatment of acute disease have potential to avoid bad outcomes among youth and working age individuals which disproportionately affect societal function. Yet the focus on specific disease outcomes limits needed systemic change, despite some efforts calling for this such as the World Health Assembly (resolution 60.22 in May 2007) supporting emergency medical services [2].

## CHANGING GLOBAL BURDEN OF DISEASE AND IMPACT ON EMERGENCY MEDICINE

As improved epidemiological data collection and modeling contribute to a better understanding of the global burden of disease, it is apparent that there is a changing nature to health needs. In addition there is a clear overlap between expected needs and emergency medicine. However, this has not resulted in expected shifts in support for emergency medicine. Particular areas of concern are: 1) increases in trauma, violence and road traffic injuries– necessitating emergent timely interventions; 2) increases in non–communicable, chronic disease, such as ischemic heart disease requiring rapid focused treatment and 3) continued high burden of communicable, maternal, neonatal disease in the world’s poorest and medically underserved places.

The authors of the global burden of disease study have called for “evidence–based health care policy” based on their predicted changes; support of emergency medical services is justified [3].

## CURRENT EFFORTS IN EMERGENCY MEDICINE DEVELOPMENT

Countries where emergency medicine is well established have a responsibility to promote worldwide emergency medicine development. Currently global health and emergency medicine overlap in a constellation of entities, which include educational projects and exchanges, specific areas of focus such as trauma and ultrasound, emergency medical services (EMS) development, and humanitarian related activities. The work of international societies of emergency medicine has made great strides in improving training and awareness [4]. Countless educational programs are making concrete improvements through education at the local level [5]. In some cases policy changes such as recognizing the specialty have been achieved, but transformation of the system has been slow and yet to be adopted on a global scale. Given the haphazard nature of global health policy making demonstrated by the historical analysis above, academic emergency medicine should be making a more cogent effort at influencing the agenda. There remains however ambiguity with regard to structural models and implementation as well as a lack of standardization with regard to education.

## EMERGENCY MEDICINE ADVOCACY: THE NEXT STEPS

### Geographic engagement

Working under the guidance of the Paris Declaration on Aid Effectiveness emergency medicine advocates should continue to work with local, national and regional societies to promote development in an ownership driven manner. Focusing as well on alignment and cooperation has been a recurrent failure in attempts to promote the specialty and training activities as evidenced by many programs working in parallel without cooperation. Development in this regard is complicated and at a minimum must involve needs assessment, the use of pre–existing resources including community involvement, local and national ownership, translation of best practices, and systemic and cultural sensitivity.

### Supporting and Influencing NGOs

Large and small NGOs represent a large proportion of global health expenditures and in following their own agendas and mandates influence policy and practical delivery of health services. Academic programs and societies of emergency medicine would also benefit from engaging the many emergency medicine NGOs in existence to improve collaborative efforts and support proven models of development. Some NGOs missions overlap well with development of emergency medical services. These NGOs can be influenced though advocacy highlighting the shared goal of improving population level health care.

### The World Health Organization

To date there has been tangential emphasis placed within the WHO on emergency medical services despite several attempts to highlight the needs and opportunities. This may change with the adoption of several programs at the WHO which have strong overlap with emergency medical services. WHO emergency medicine related areas include, trauma care, hospital and health system disaster preparedness and hospital and essential Medicines standards. While these issues fall under the rubric of emergency medicine, codifying it as such will be necessary to take a next step forward in influencing policy toward emergency medicine development.

### Philanthropy

Philanthropy has long held a disproportionate influence on global health policy. Large philanthropic organizations sway policy not only through simple financial means, but through collaboration with grant recipients and in the extraordinary publicity they garner. Philanthropic foundations are often very closely tied to industry and exist in a complex environment with competing missions and conflicts of interest resulting in less than transparent policy making. Very few organizations have any explicit focus on emergency medical services and vertical programing is still common. However, emergency medicine can and should be appealing to even those in philanthropic positions of power, as they and their staff are often on the ground in settings without any reasonable emergency medical care. Sadly, as is the case in all situations where medical care is not reliable, avoidable medical disasters do occur, even to those visiting for humanitarian reasons. Highlighting the supporting role emergency medical care can provide to any population (obstetrical, pediatric) or disease entity (malaria, HIV) may also gather support.

### Research and public relations

Emergency medicine can do a lot to support its own development with regards to building a clear and well supported message of improved outcomes and cost efficiency. To date, clear evidence of improved population level gains by the institution of comprehensive emergency medical services has not been established, nor has clear cost-effectiveness or a universally successful model of implementation. Citing particular emergency services entities that have proven cost-effective such as defibrillators, so far has not resulted in systemic change [6].

## CONCLUSION

The call for a global movement towards improved emergency medicine services, while not new, has failed to produce the desired results. This, combined with the window of opportunity due to recent focus on global health, makes re–addressing and re–focusing efforts towards a cogent policy a great priority and opportunity.

While best practices and implementation pathways are not clear, collaboration, assessment and needs driven policy, with locally and national involvement and sensitivity are part of best practices. Persuading global level policy makers to accept that provision of emergency medical services is an essential part of health system development should be the highest priority of those working towards emergency medicine development.
